# Real Time Anti-*Toxoplasma gondii* Activity of an Active Fraction of *Eurycoma longifolia* Root Studied by *in Situ* Scanning and Transmission Electron Microscopy

**DOI:** 10.3390/molecules17089207

**Published:** 2012-08-02

**Authors:** Nowroji Kavitha, Rahmah Noordin, Chan Kit-Lam, Sreenivasan Sasidharan

**Affiliations:** 1 Institute for Research in Molecular Medicine (INFORMM), Universiti Sains Malaysia, Pulau Pinang 11800, Malaysia; 2 School of Pharmaceutical Sciences, Universiti Sains Malaysia, Pulau Pinang 11800, Malaysia

**Keywords:** *Toxoplasma gondii*, *Eurycoma longifolia*, transmission electron microscope, scanning electron microscope, toxoplasmosis

## Abstract

The inhibitory effect of active fractions of *Eurycomalongifolia* (*E. longifolia*)root, namely TAF355 and TAF401, were evaluated against *Toxoplasma gondii (T. gondii).* In our previous study, we demonstrated that *T. gondii* was susceptible to TAF355 and TAF401 with IC_50_ values of 1.125 µg/mL and 1.375 µg/mL, respectively. Transmission (TEM) and scanning electron microscopy (SEM) observations were used to study the *in situ* antiparasitic activity at the IC_50_ value. Clindamycin was used as positive control. SEM examination revealed cell wall alterations with formation of invaginations followed by completely collapsed cells compared to the normal *T. gondii* cells in response to the fractions. The main abnormality noted via TEM study was decreased cytoplasmic volume, leaving a state of structural disorganization within the cell cytoplasm and destruction of its organelles as early as 12 h of treatment, which indicated of rapid antiparasitic activity of the *E. longifolia* fractions. The significant antiparasitic activity shown by the TAF355 and TAF401 active fractions of *E. longifolia* suggests their potential as new anti-*T. gondii* agent candidates.

## 1. Introduction

*Eurycomalongifolia* Jack, from the Simaroubaceae family and identified locally as ‘Tongkat Ali’ or ‘Pasakbumi’ has been commonly prescribed in traditional medicine as a febrifuge and a remedy for dysentery, glandular swelling and fever [1,2]. *E. longifolia* is found in primary and secondary, evergreen and mixed deciduous forests in Burma, Indochina, Thailand, Malaysia, Sumatra, Borneo and The Philippines. It is popularly sought after as a single or an essential component for the treatment of fevers, aches, sexual insufficiency and also as a health supplement. Traditional medicinal users usually take a decoction of the roots in water as a health tonic and anti-stress remedy. Extracts derived from the roots of this plant were also found to demonstrate activity when evaluated with the sarcoma 180 model [3]. Moreover, anti-malarial [4,5,6,7,8] and cytotoxic [9,10,11] activities were also reported being linked to the presence of quassinoids, squalene derivatives, biphenyl-neolignans, tirucallane-type triterpenes, canthine-6-one and carboline alkaloids. Specially, three quassinoids, eurycomanone, its 13 α-(21)-epoxy and 13,21-dihydro analogues were identified as having greater anti-plasmodial activity [12].

One of the common infections in tropical and subtropical climates is toxoplasmosis caused by *Toxoplasma gondii*. It is one of the most widespread protozoan parasites, chronically infecting approximately 30% of the global human population [13]. *T. gondii* causes severe neurological deficits in immunosuppressed patients (such as those with AIDS) and lymphadenopathy in healthy adults. It can cross the placenta (generally in women with no or low antibody levels) and cause congenital infections characterized by intra-cerebral calcifications, chorioretinitis, hydrocephaly or microcephaly, and convulsions [14]. From this perspective, new agents from natural resources that can inhibit the growth of *T. gondii* are greatly needed and would enhance the effectiveness of therapy. Our previous study showed that two active fractions of *E. longifolia* root, namely TAF355 and TAF401, possessed a good antiparasitic activity against *T. gondii* with IC_50_ values of 1.125 µg/mL and 1.375 µg/mL, respectively [15]. The median inhibition concentration (IC_50_) value refers to the concentration of the fraction necessary to inhibit at 50% of the control values. The present investigation demonstrates the antiparasitic activity of the *E. longifolia* root fractions at the ultra-structural level through TEM and SEM observations at IC_50_ concentration of the fractions. Advances in microscopy technique to observed at ultrastructural level of cells morphology enhancing understanding of *in situ* antiparasitic activity observation. The microscopy method enables *in situ* observations of the effect of anti-parasitic agents on the organisms [16]. In this study TEM and SEM technique was used to observe the suppression of *T. gondii* growth by clindamycin (positive control), TAF 355 and TAF 401.

## 2. Results and Discussion

The rapidly increasing prevalence of toxoplasma encephalitis in AIDS patients has highlighted the need for more effective therapies and for alternative drugs for the considerable number of patients (up to 60%) who develop allergic reactions or serious side effects during standard therapy with a sulfonamide and trimethoprim or pyrimethamine [17]. Therefore, the anti-parasitic activity and prevention of *T. gondii* infection by local herbal products were emphasized in our present work for the reasons that the management of *T. gondii* infections faces a number of problems, including a limited number of effective anti-*T. gondii* agents; toxicity of the available drugs; resistance of *T. gondii* to commonly used drugs and lack of cost effective anti-*T. gondii* agents. Among the different strategies which have been identified to defeat drug resistance, the investigation of new and effective natural products exhibiting antiparasitic activity may possibly play an important role to overcome drug resistance and the side effects. 

In this paper, we were especially interested in the morphogenesis of the *T. gondii* cells challenged by active fractions from roots of *E. longifolia*. *In vitro* observation of anti-*T. gondii* activity of the active fractions from root of *E. longifolia*, was further evaluated in this study by *in situ* microscopic methods to examine the possible mechanisms of action in *T. gondii*. The morphological observation of *T. gondii* cells treated with plant extracts by using electron microscope is considered a gold standard technique to study the *in situ* real time anti-*T. gondii* activity. The SEM and TEM techniques are useful compared to several other microscopic methods as they are three-dimensional and almost the whole cell of the specimen is sharply focused [16]. Since the SEM and TEM allow visualization of surface phenomena under high magnification from a three-dimensional perspective, in this study, SEM and TEM observations were utilized to study the anti-*T. gondii* activity of active fractions from roots of *E. longifolia*.

**Figure 1:**
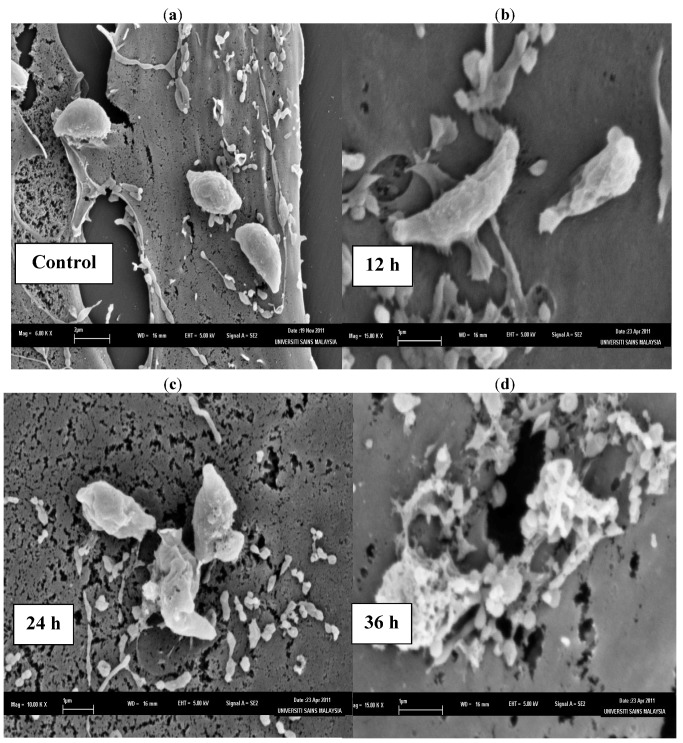
SEM micrograph of the untreated and clindamycin treated cells of *T. gondii* after different time intervals. (**a**) Untreated cells, (**b**) 12 h of exposure, (**c**) 24 h treated cells, (**d**) 36 h of exposure.

The SEM photomicrographs of the untreated and treated cells of *T. gondii* at various times of exposure to clindamycin are shown in Figure 1. Untreated cells (Figure 1a) showed crescent-shaped cells and possess two differently structured poles; one is more round, the other is more pointed and probably the site of the conoid. The surface of the untreated tachyzoites is smooth, regular and homogeneous. After 12 h of exposure (Figure 1b), a mild effect of the clindamycin on *T. gondii* was observed, with shape irregularities compared to the untreated control cells. The 24 h treated cells (Figure 1c) show distorted cell membranes as compared to the normal *T. gondii* cells, followed by collapsed cells. After 36 h of exposure (Figure 1d), completely collapsed and cavitated cells with cytoplasmic eruption were seen. It was believed that at this stage, the cells had lost their metabolic functions completely. 

**Figure 2:**
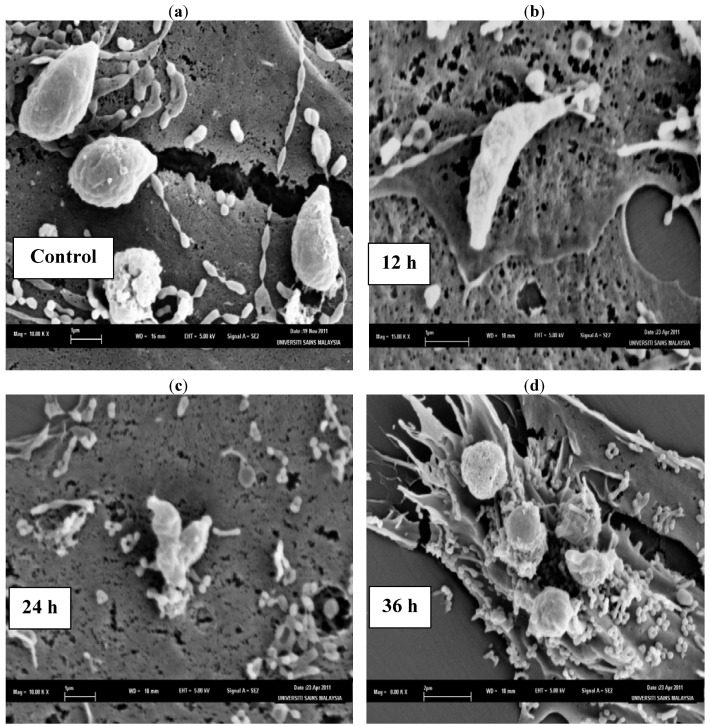
SEM micrograph of the untreated and TAF355 treated cells of *T. gondii* after different time intervals. (**a**) Untreated cells, (**b**) 12 h of exposure, (**c**) 24 h treated cells, (**d**) 36 h of exposure.

The SEM photomicrographs of the untreated and treated cells of *T. gondii* at various times of exposure to TAF355 are shown in Figure 2. Untreated cells (Figure 2a) showed crescent-shaped cells and possess two differently structured poles; one is more round, the other is more pointed and probably the site of the conoid. The surface of the untreated tachyzoites is smooth, regular and homogeneous. After 12 h of exposure (Figure 2b), a mild effect of the TAF355 and elongated cells of *T. gondii* were observed with irregular shapes compared to the untreated control cells. The 24 h treated cells (Figure 2c) show distorted cell membranes with the formation of invaginations as compared to the normal *T. gondii* cells. After 36 h of exposure (Figure 2d), completely collapsed cells with cytoplasmic eruption were observed. It was believed that at this stage, the cells had lost their metabolic functions completely. Huge amounts of vesicular material were equally found scattered in between the *T. gondii* cells, was most likely derived from broken cells and presented membrane-limited cytoplasmic residues.

**Figure 3:**
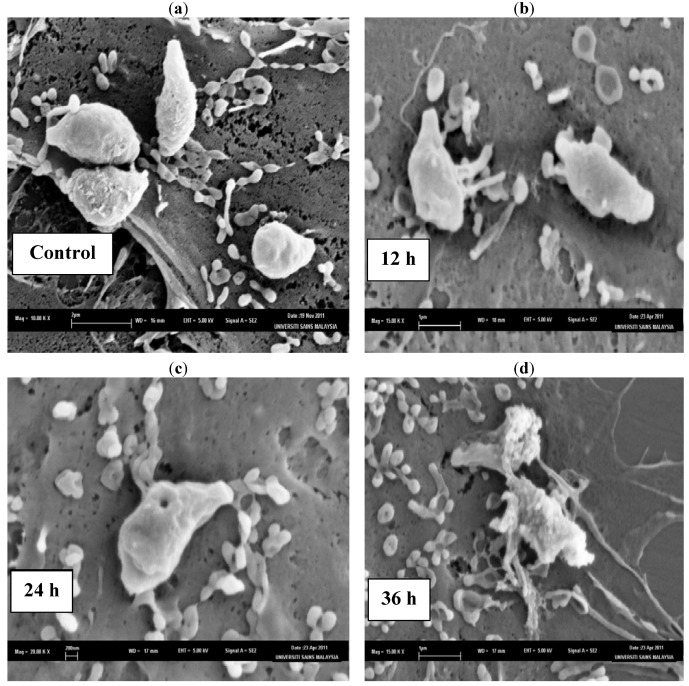
SEM micrograph of the untreated and TAF401 treated cells of *T. gondii* for different time interval. (**a**) Untreated cells, (**b**) 12 h of exposure, (**c**) 24 h treated cells, (**d**) 36 h of exposure.

The SEM photomicrographs of the untreated and treated cells of *T. gondii* at various times of exposure to TAF401 are shown in Figure 3. Untreated cells (Figure 3a) showed crescent shapes and possess two differently structured poles; one is more round, the other is more pointed and probably the site of the conoid. The surface of the untreated tachyzoites is smooth, regular and homogeneous. After 12 h of exposure to TAF401 (Figure 3b), a mild effect with irregular *T. gondii* cell shapes was observed, compared to the untreated control cells. The 24 h treated cells (Figure 3c) had a rough appearance with holes compared to the untreated control cells, with the formation of invaginations. After 36 h of exposure to TAF401 (Figure 3d), severe alterations of the cell wall with the formation of invaginations were seen and followed by the complete collapse of the cells. The *T. gondii* cells lost their symmetrical appearance, they were completely deformed, and deep wrinkles were observed. Cell wall invaginations were also noticed. Leakage of cellular content could be noticed.

**Figure 4:**
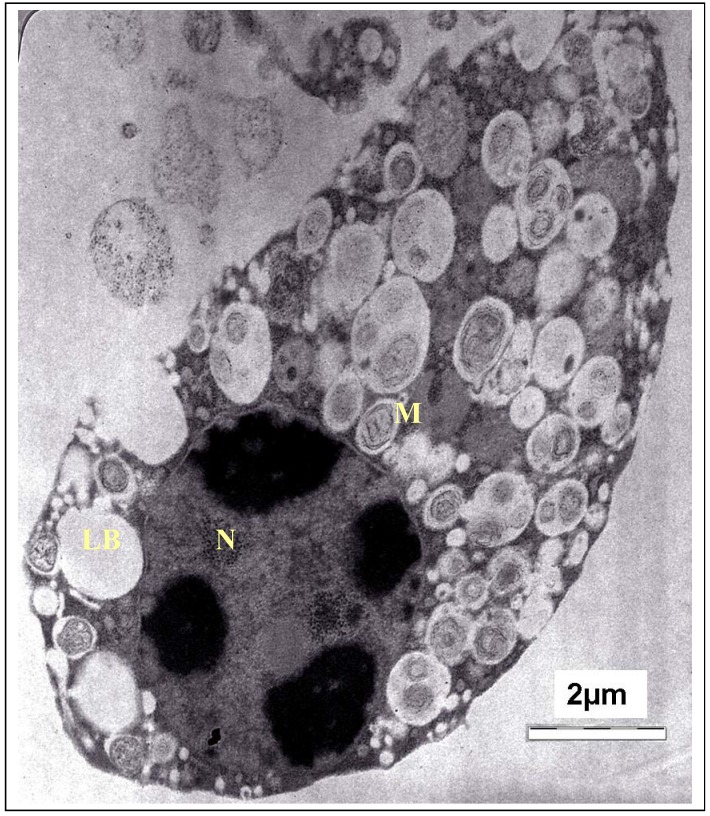
TEM micrograph of the control cells of *T. gondii*. M, mitochondrion; N, nucleus; LB, Lipid body.

From the SEM findings, it can be suggested that the *T. gondii* cells had undergone some distinct morphological and cytological alterations. Further evidence of these changes was obtained by TEM observations on similarly treated *T. gondii* cells. As expected, the TEM observation also reaffirmed the disorganization of *T. gondii* cells and destruction of their organelles. The TEM photomicrograph of the untreated cells of *T. gondii* is shown in Figure 4*.* As the fine structure of tachyzoites of *T. gondii* has been reported by numerous investigators, only a brief description of the parasite is presented here. It showed a typically structured cytoplasm with various organelles. The tachyzoite is often crescent shaped, approximately 2 by 6 mm, with a pointed anterior (conoidal) end and a rounded posterior end. Ultrastructurally, the tachyzoite consists of various organelles and inclusion bodies including a pellicle (outer covering), apical rings, polar rings, conoid, rhoptries, micronemes, micropore, mitochondrion, subpellicular microtubules, endoplasmic reticulum, Golgi complex, ribosomes, rough and smooth endoplasmic reticula, micropore, nucleus, dense granules, amylopectin, granules (which may be absent), and a multiple-membrane-bound plastid-like organelle which has also been called a Golgi adjunct or apicoplast [18]. 

**Figure 5:**
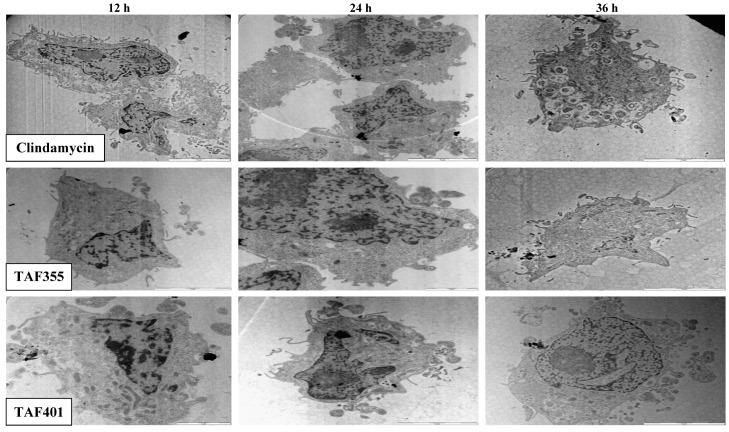
TEM micrographs of the treated treated cells of *T. gondii* for different time intervals.

Figure 5 shows the TEM photomicrographs of the longitudinal and transverse sections of the clindamycin, TAF 355 and TAF 401 treated cells of *T. gondi* at various time intervals. After 12, 24 and 36 h of exposure to clindamycin, TAF 355 and TAF 401, the cells exhibited notable alterations in the cell cytoplasm. 

The cytoplasmic volume decreased, leaving a state of structural disorganization within the cell cytoplasm and destruction of its organelles. It shows that the parasite cells collapsed and lysed. Ultrastructural effects of clindamycin, TAF 355 and TAF 401 were detected as early as 12 h after the start of treatment. General views of thin sections showed completely destroyed parasites after treatment of infected cells with IC_50_ concentration of clindamycin, TAF 355 and TAF 401, even at 12 h of treatment. This implied that the TAF 355 and TAF 401 fractions have a significant effect on the *T. gondii* cells or their membranes as early as 12 h after treatment which indicated a rapid antiparasitic activity of these *E. longifolia* fractions. Of further interest are the inconsistent findings between SEM and TEM observations concerning the viability of the *T. gondii* cells, *i.e.*, completely collapsed cells only observed after 36 h of treatment with SEM examination, contradictorily upon TEM examination it was found to be completely necrotic even at 12 h of treatment. This means that lytic changes in cell cytoplasm probably appear faster and do not affect the cell walls as observed in this study.

The microscopic examination of *T. gondii* using SEM showed that the cells treated with TAF 355 and TAF 401 appeared irregular in shape with cell wall modifications and clear depressions on the cell surface with holes. Also, TEM observation showed irregular cell shape, unclear periplasm and dense cytoplasm without differentiated features. Such modifications may be related to the interference of the *E. longifolia* active fractions with enzymatic reactions of metabolism activities of *T. gondi* which affects parasite morphogenesis and growth, which deserves further research. Actually, the antiparasitic action of *E. longifolia* active fractions may happen in two steps. The first step involves the passive entry of the active fractions into the plasma membrane in order to hit the metabolic activities of *T. gondi* in the cytoplasm region. The second stage is the accumulation of *E. longifolia* active fractions in the cytoplasm resulting in inhibition of cell growth. With regard to the mechanism of action for TAF 355 and TAF 401 against *T. gondii* in the cytoplasm region, we hypothesize that TAF 355 and TAF 401 may produce intracellular oxidative stress by an indirect mechanism [19]. Mitochondria are the largest source of reactive oxygen species (ROS) within cells [20]. Moreover, uncontrolled superoxide flashes in mitochondria contribute to global oxidative stress, playing a key role in hypoxia/reoxygenation injury in cells [21]. This model provides a rational explanation for why TAF 355 and TAF 401 inhibit *T. gondii* growth.

## 3. Experimental

### 3.1. Plant Collection

The roots of *E. longifolia* Jack were identified and purchased from Perak, Malaysia by a pharmaceutical company, Hovid Berhad, Ipoh. A voucher specimen (No. 785-117) was deposited in Penang Botanical Garden, Penang, Malaysia.

### 3.2. Preparation of Fraction

The air-dried powdered root of *E. longifolia* was extracted with 6 × 4 L of 95% methanol for 6 days at 60 °C. The combined methanol extract was then evaporated to dryness to yield a dark brown residue. Subsequently, this dark brown residue was chromatographed on a AB-8 resin column with several alcohol/water mixtures to yield four fractions (Fr 1–4) identified as alcohols layers, water layer and residue layers. The four fractions were concentrated under vacuum and then resuspended in water and partitioned successively with saturated *n*-butanol to yield several sub-fractions. Successive column chromatography using silica gel and centrifugal thin-layer chromatography of the sub-fractions with various CHCl_3_-methanol mixtures yielded the desired two active sub-fractions fractions (TAF 355 and TAF 401). The fractions that contained TAF 355 and TAF 401 were identified by TLC comparison. Two active fractions (TAF 355 and TAF 401) were used in this study. The Roswell Park Memorial Institute 1640 (RPMI-1640) medium was used as the solvent for preparation of different dilutions of the plant active fractions. The dried fractions were then redissolved in RPMI-1640 medium to yield solution containing IC_50_ of fraction per milliliter. 

### 3.3. Toxoplasma Gondii Strain

The experimental procedures relating to the animals were authorized Universiti Sains Malaysia Ethical committee (USM/PPSF50(003)JLD2) before starting the study and were conducted under the internationally accepted principles for laboratory animal use and care. Tachyzoites from the virulent RH strain of *T. gondii* were maintained by intraperitoneal passages in Swiss albino mice and collected in phosphate-buffered saline (PBS), pH 7.2, at 3–4 day intervals. The ascites fluid obtained from infected mice was centrifuged at 200 ×g for 10 min at room temperature to remove host cells and debris. The supernatant, which contained the parasites, was collected and centrifuged at 1000 ×g for 10 min. The pellet was washed with PBS, pH 7.2 and then in RPMI medium without foetal bovine serum (FBS). The parasites were used within 30–40 min of their removal from the mice peritoneal cavity and the viability was evaluated using the trypan blue dye-exclusion test.

### 3.4. Host Cells

The results of our previous study indicated that *E. longifolia* fractions did not have a significant toxic effect on Vero cell growth, and *E. longifolia* fractions can be used safely for the anti-*Toxoplasma* assay [22]. The Vero cell line was initiated from kidney of a normal adult African green monkey on March 27th, 1962, by Yasummura and Kawakita at the Chiba University, Japan (American Public Health Association, 1992). Vero cells were maintained in RPMI-1640 medium supplemented with 10% FBS, glutamine (2 raM), penicillin (100 units/mL) and streptomycin (100 μg/mL). The cells were cultured at 37 °C in a humidified 5% CO_2_ incubator. 

### 3.5. Ultrastructural Observations

The general structure of control and drug-treated tachyzoites of *T. gondii* has been previously described [23].

#### 3.5.1. Scanning Electron Microscopy (SEM) Observation of the Tachyzoites in Vero Cells

Vero cells were cultured on a glass cover slip in a 35 mm cell culture dish until confluent, and then infected with 1 × 10^4^ tachyzoites/dish. After incubation for 4 h, the monolayers were washed with 0.1 M phosphate buffer (pH 7.4). Subsequently, TAF355, TAF401 and clindamycin in RPMI-1640 medium (at IC_50_ concentration) or RPMI-1640 medium as a solvent control were added to the monolayers. The glass cover slips were taken from the dishes at 0, 12, 24 and 36 h after adding the drugs. All the glass cover slips were washed with Hanks balanced salt solution (HBSS; Gibco Inc., Grand Island, NY, USA) and were fixed for SEM with Macdowell-Trump fixavative prepared in 0.1 M phosphate buffer (pH 7.2). SEM analyses were performed on cover slips by locating on double-stick adhesive tabs on a planchette and the planchette was placed in a Petri plate. In a fume hood, a vial cap containing 2% osmium tetroxide in water was placed in an unoccupied quadrant of the plate. After being covered, the plate was sealed with parafilm, and vapor fixation of the sample proceeded for 1 h. Once the sample was vapor fixed, the planchette was plunged into slushy nitrogen (−210 °C) and transferred on to the “peltier-cooled” stage of the Freeze Dryer (Emitech K750), and freeze drying of the specimen was proceeded for 10 h. Finally, the freeze dried specimen was sputter coated with 5–10 nm gold before viewing in the SEM (LEO SUPRA 50 VP Field Emission SEM, Carl Zeiss, Oberkochen, Germany) operating at 15 kV at various levels of magnification.

#### 3.5.2. Transmission Electron Microscopy (TEM) Observation of the Tachyzoites in Vero Cells

For TEM, confluent lawns of tachyzoites were treated with TAF355, TAF401 and clindamycin in RPMI-1640 medium (at IC_50_ concentration) or RPMI-1640 medium as a solvent control. Parasites were harvested at 0, 12, 24 and 36 h after adding the drugs and resuspended in 1 mL ice-cold PBS, transferred to 1.5 mL Eppendorf tubes, and centrifuged (5,000 ×g, 5 min, 4 °C). Pellets were resuspended in 100 mM cacodylate (pH 7.3) containing 2.5% glutaraldehyde and fixed overnight at 4 °C. Pellets were then washed three times in 100 mM cacodylate buffer and post fixed in 100 mM cacodylate containing 1% OsO_4_ for 2 h. Pellets were then washed three times in distilled water and directly dehydrated in an ethanol series (50%, 70%, 90%, 3 × 100%). Subsequently, the samples were embedded in Epon 820 resin [24]. The resin was polymerized at 65 °C over a period of 48 h. Ultrathin sections were cut on a ultramicrotome and loaded onto 300-mesh copper grids (Plano GmbH, Marburg, Germany). Staining with uranyl acetate and lead citrate was performed as described previously [24]. Finally, grids were viewed on a transmission electron microscope (LIBRA 120-ZEISS, Oberkochen*,* Germany) operating at 5.00 kV.

## 4. Conclusions

In conclusion the active fractions from *E. longifolia* designated as TAF 355 and TAF 401 have potent and selective antiproliferative activity against *T. gondii* tachyzoites. Hence, *T. gondii* infection can be treated with the fractions, as the IC_50_ values of TAF 355 and TAF 401 for this parasite was found to be only of 1.125 µg/mL and 1.375 µg/mL, respectively. The *E. longifolia* fractions have never been evaluated for *in situ* anti-*T. gondii* activity before, and thus, they have been shown for the first time in this study to have *in situ* anti-*T. gondii* activity. Based on the present results and previously reported studies, the efficient killing activities of these *E. longifolia* fractions show the potential of these fractions as a novel toxoplasmacidal drug. Therefore, further purification of active compound(s) from *E. longifolia* fractions and *in vivo* studies in an animal model can be suggested on the basis of the present study. 
